# *Peganum Harmala *L*. *Extract Reduces Oxidative Stress and Improves Symptoms in 6-Hydroxydopamine-Induced Parkinson*’*s Disease in Rats

**Published:** 2016

**Authors:** Maryam Rezaei, Sima Nasri, Mehrdad Roughani, Zeinab Niknami, Seyed Ali Ziai

**Affiliations:** a *Razi Herbal Medicines Research Center, Lorestan University of Medical Sciences, Khoramabad, Iran. *; b *Department of Biology, Payam Noor University, Tehran, Iran. *; c *Department of Pharmacology, School of Medicine, Shahed University, Tehran, Iran. *; d *Department of Pharmacology, School of Medicine, Shahid Beheshti University of Medical Sciences, Tehran, Iran*

**Keywords:** *Peganum harmala*, Parkinson’s disease, 6-hydroxydopamine, angiotensin converting enzyme, rat

## Abstract

Parkinson’s disease is one of the most common neurodegenerative disorders. There are many documents about the effects of oxidative stress in Parkinson’s disease etiology. Angiotensin II activates NADPH dependent oxidases and causes superoxides formation. *Peganum harmala* L. extract, which has angiotensin converting enzyme (ACE) inhibitory effect, is considered to evaluate oxidative stress inhibition and Parkinson's disease improvement.

Male rats weighting 200-250 g were divided into 5 groups: Control, Neurotoxin (injection of 6-hydroxydopamine into left hemisphere substantia nigra), *Peganum harmala's *seeds aqueous extract (10 mg/kg) and captopril (5 mg/kg). *Peganum harmala *and captopril were injected intraperitonealy -144, -120, -96, -72, -48, -24, -2, 4 and 24 h relative to 6-hydroxydopamine injection time. Muscle stiffness, apomorphine induced unilateral rotation, amount of brain's protein oxidation and lipid peroxidation, ACE activity and histology of substantia nigra were assayed in all groups.

*Peganum harmala *improved Muscle stiffness and one-direction rotation behavior significantly. It also reduced brain's lipid and protein oxidation levels in neurotoxin-injected rats significantly. In *Peganum harmala *group compared to control group, brain's ACE activity was significantly inhibited. In histological study,* Peganum harmala* prevented degeneration of dopaminergic neurons, too.

In conclusion, aqueous extract of *Peganum harmala *could prevent symptoms and reduced oxidative stress markers in rats with Parkinson’s disease induced by 6-hydroxydopamine.

## Introduction

Parkinson's disease (PD) is the most prevalent neurodegenerative disease after Alzheimer's disease (1). In PD the basal ganglia cells and substantia nigra (SN) cells are destroyed and then the level of dopamine is decreased (2). 

Increasing evidence showed the role of oxidative stress as a pathogenic factor in PD (3). Oxidative stress is the release of reactive oxygen species (ROS). Some of the most important causes of oxidative stress are aging, genetic factors, metals, changing in vital macromolecules, diet, lifestyle, free radicals, poisons, and Angiotensin II (4, 5). 

Angiotensin converting enzyme (ACE) converts angiotensin І to angiotensin П. Angiotensin П activates oxidases related to NADPH and creates superoxide (4, 6). SN and striatum have higher ACE activity compared to other brain regions, and AT1 receptors co-localize with nigral dopamine neurons (7-9). Angiotensin П creates ROS by AT1 receptors and destroys the dopaminergic neurons, so manipulation of renin angiotensin system (RAS) may be effective in treatment of PD (10). ACE inhibitors like perindopril and captopril and angiotensin II receptor blockers like losartan had neuroprotective effect in the striatum and the SN in rats receiving 6-hydroxydopamine (6-OHDA) (11-13).

Aqueous extract of* Peganum harmala's *seeds (AEPHS) showed ACE inhibitory effect *in-vitro *(14, 15). This study examined possible anti-Parkinsonism effect of AEPHS compared to captopril in 6-OHDA induced PD in rats.

## Experimental

*Animals*

Male Wistar rats 200-250 g, were allocated in the 4 groups. In each group 6 rats were killed for biochemical tests and measurement of ACE activity 24 h after the 6-OHDA neurotoxin injection and the 6 others were kept for behavioral tests and histopathological study for 2 weeks after the 6-OHDA injection. All experiments were performed in accordance with the National Institutes of Health Guide for the Care and Use of Laboratory Animals.

1) Toxin group: their left hemisphere’s substantia nigra (SN) was destroyed by 6-OHDA. 

2) Control group: or sham operated group, was received normal saline instead of AEPHS or 6-OHDA.

3) *Peganum harmala* group: AEPHS (10mg/kg) was injected i.p -144, -120, -96, -72, -48, -24, -2, 4 and 24 h relative to injection time of 6-OHDA into SN.

4) Captopril group: captopril (5mg/kg) was injected i.p -144, -120, -96, -72, -48, -24, -2, 4 and 24 h relative to injection time of 6-OHDA into SN.

*Aqueous extraction of* *Peganum harmala* *seeds*

100 grams of dried plant's seeds was poured into 1 liter boiling water in a beaker and kept in room temperature for 2 h. After that the solution was filtered and freeze-dried.

*Parkinsonism induction*

Each rat was anaesthetized by i.p injection of 100 mg/kg ketamin and 5 mg/kg xylazine and then his head was fixed on stereotaxic device (Stoelting, USA). Stereotaxic parameters for SN: AP: -4.8 mm to brigma, ML (left): 2 mm, DV: -8.3 mm from the surface of scalp by Watson & Paxinos atlas. 4μL of toxin (2mg/mL 6-OHDA with 0.1% vitamin C in normal saline) was injected by Hamilton syringe at a rate of 1 μl/min (16).

*Rotation test*

We tested rats' unidirectional rotation test induced by apomorphine hydrochloride (2.5 mg/kg) in PD rats. Whole (right-sided minus left-sided) rotation in a cylinder box (33 cm diameter, 35 cm height) was measured in an isolated room in a 60 min. period.

*Murprogo's test*

This is a method to measure muscle stiffness (17), by laying the animal on a flat surface the rat received a score of 0.5 if it did not move when touched. After that the right paw of the rat was laid on the edge of a box with 3 cm height. If the animal did not take its paw off after 10 sec, it received a score of 0.5. The same method was used for the left hand. In the next step, only the right paw of the rat was placed on the edge of a box with a height of 9 cm. If the rat did not take its paw off after 10 s, it received a score of 1. The last step was repeated for the left hand of the rats. The sum of the scores of movement on the floor and movement of hands while being hanged on the edge of boxes with 3 cm and 9 cm heights was 3.5.

*ACE enzyme activity in serum blood and brain tissue homogenate*

Brains were kept in -80ºC freezers until analysis time. Brain tissue was homogenated and 10 μl of homogenate was incubated with 40 μl substrate (hippuryl histidyl leucine) in a thermo-mixer (eppendorf- MTP model) for 30 min. in 37˚C and 300 rpm. After that, 150 μl phosphoric acid (5M) was added to each well to stop the reaction. 20 μl of the reactant mixture was injected into HPLC (Shimadzu, pump: LC-10ADVP, control system: SCL-10AVP, detector: SPD-10AV) and area under the curve of hippuric acid (enzyme product) was detected in 228 nm with 1 mL/min flow rate of mobile phase consisting of 1: 1 methanol: KH_2_PO_4_ 0.1M, pH = 3. One unit of enzyme activity was defined as: 1 nmol of hippuric acid produced in one mg of brain tissue protein in one min in 37˚C. 

*Lipid peroxidation*

Lipid peroxidation was tested by complex formation between malondialdehyde and thiobarbitoric acid. Thiobarbitoric acid reactive substances (TBARS) were measured by spectrophotometer at 532 nm (5). 

Protein concentration was measured by Bradford method with BSA (bovine serum albumin) as standard (18).

*Protein oxidation*

Protein oxidation was tested by measuring the concentration of carbonyl groups of proteins. Carbonyl group content of protein was assayed by spectrophotometer at 370 nm (19). Carbonyl group concentration was calculated based on e = 22 mM^-1^cm^-1^. 

*Histology examination*

After decapitation, 5 to 8 cut of SN was processed for the number of dopaminergic neurons. We counted Nissl-stained dopaminergic neurons in the substantia nigra pars compacta and substantia nigra pars reticulate region in left and right hemispheres at 200x zoom. 

*Chemicals used in experiments*

1, 1, 3, 3-Tetraethoxy propane, 2, 4- Dinitrophenyl Hydrazine, Apomorphine hydrochloride, Cresyle violet acetate, Guanidine hydrochloride, Hippuryl-His-Leu, Streptomycin sulfate, Tritonx-100, Desferrioxamine, and 6-hydroxydopamine were purchased from Sigma-Aldrich. Ketamine, Xylazine, Magnesium acetate tetrahydrate, Sucrose, Thiobarbituric acid, and Trichloro acetic acid were obtained from Merck. 

*Statistical analysis*

 Because of failure of normality distribution, we used non parametric Kruskal-Wallis test, and comparisons between 2 groups were made by Mann-Whitney U test. All analysis were done by IBM SPSS Statistics ver. 20.

## Results

*Number of unilateral rotation*

**Figure 1:**
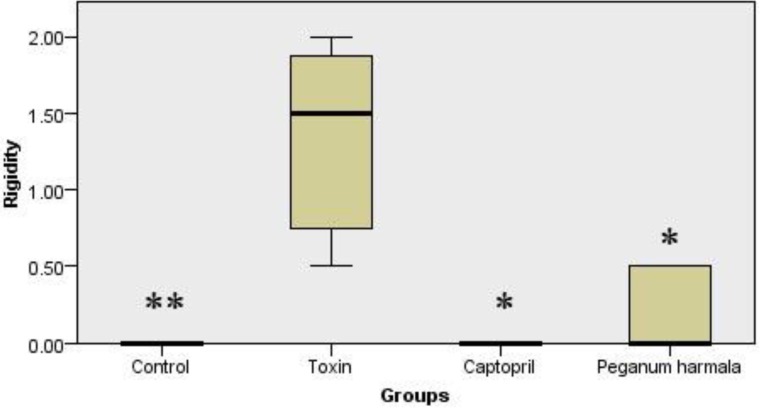
Number of left handed rotation in one h period in the study groups. Kruskal-

Left handed rotation in 1 h period in toxin group was significantly higher than the control and two treatment groups (Figure1).

Wallis test showed a significant difference (p = 0.002) between study groups.

*p < 0.05 compared to toxin group tested by Mann-Whitney U test.

**p < 0.01 compared to toxin group tested by Mann-Whitney U test.

*Murprogo's test*

**Figure 2:**
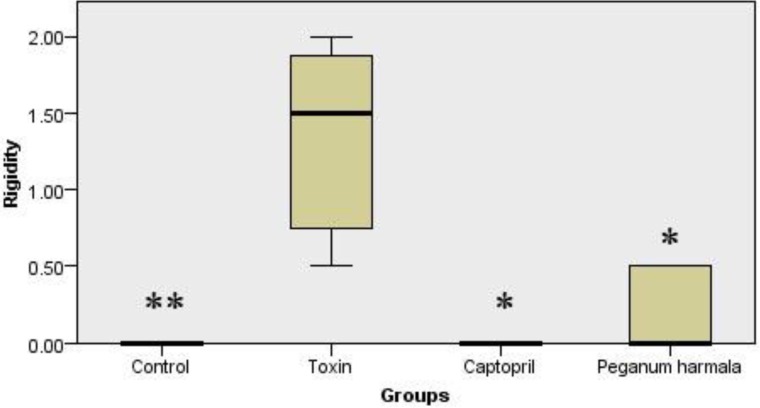
Murpogo's test for stiffness evaluation in rats. Kruskal-Wallis test showed a significant (p = 0.002) difference between study groups

Rigidity was significantly higher in toxin group compared to other groups (Figure 2).

*p < 0.05 compared to toxin group tested by Mann-Whitney U test.

**p < 0.01 compared to toxin group tested by Mann-Whitney U test.

*Lipid peroxidation*

**Figure 3:**
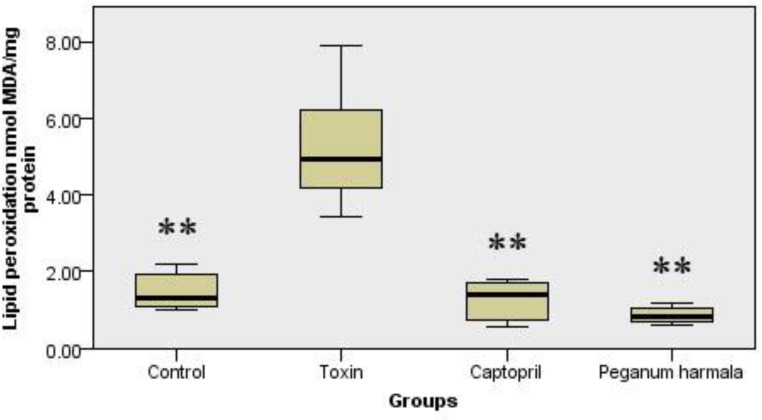
Malondialdehyde concentration of brain in study groups. Kruskal-Wallis test showed a significant (p = 0.001) difference between study groups

Malondialdehyde production as a lipid peroxidation index was significantly higher in toxin group than other groups (Figure 3).

*Protein oxidation*

**Figure 4:**
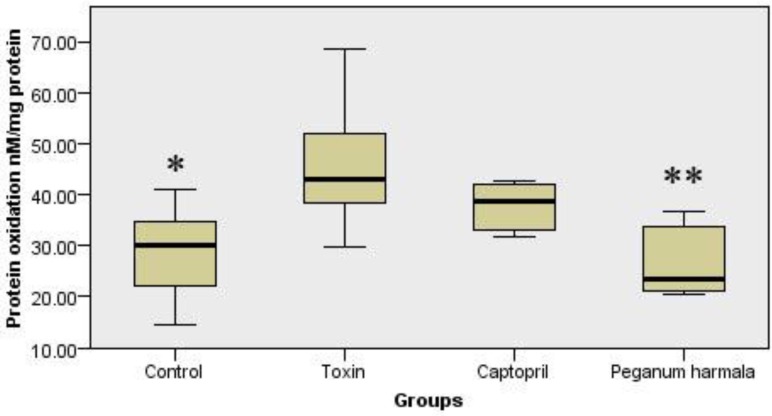
Carbonyl group content was studied as a protein oxidation marker in the study groups. Kruskal-Wallis test showed a significant (p = 0.012) difference between study groups.

Protein oxidation in *Peganum harmala* group was significantly lower than toxin group (Figure 4).

*p < 0.05 compared to toxin group tested by Mann-Whitney U test.

**p < 0.01 compared to toxin group tested by Mann-Whitney U test.

*Brain ACE activity*

**Figure 5:**
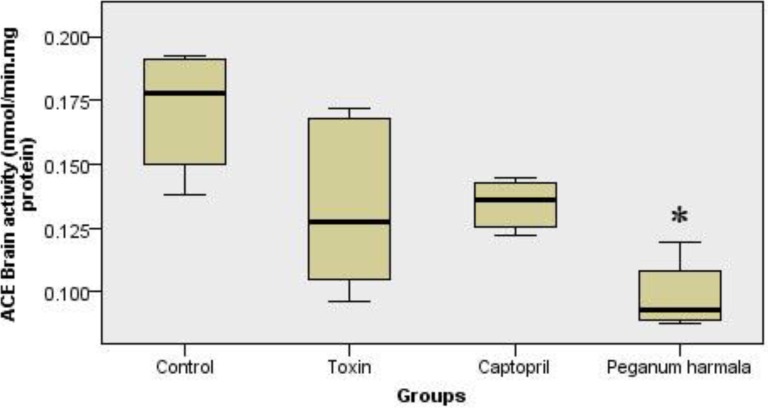
ACE activity in brain (nanomole hippuric acid produced per minute per mg brain protein content in 37˚C) in *peganum harmala* group was significantly lower than toxin group. Kruskal-Wallis test showed a significant (p = 0.004) difference between study groups.

*Peganum harmala* significantly inhibited ACE activity in the brain compared to toxin group (Figure 5).

*p < 0.05 compared to toxin group tested by Mann-Whitney U test.

*Histology examination*

**Figure 6:**
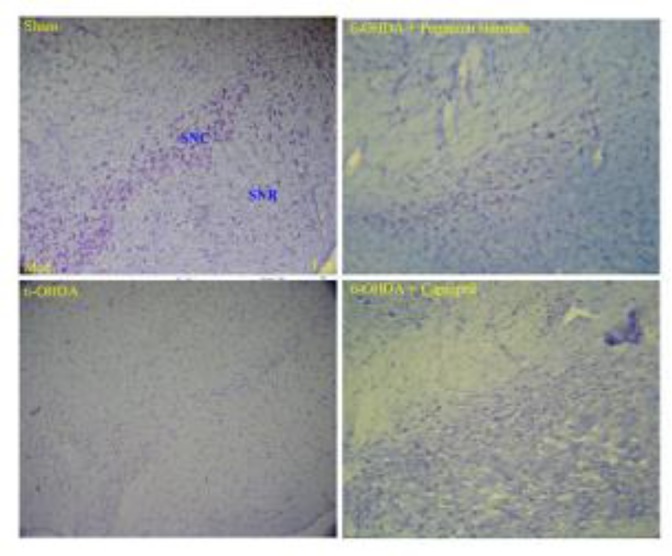
Photographs of typical coronal section through the midbrain showing Nissl-stained dopaminergic neurons in the control (sham, upper left hand), toxin (6-OHDA, lower left hand), *Peganum harmala* (6-OHDA + *Peganum harmala,* upper right hand), and captopril (6-OHDA + captopril, lower right hand) groups. SNC: substantia nigra pars compacta, SNR: substantia nigra pars reticulate

There were no significant differences in the number of dopaminergic neurons in left and right hemispheres in control group, but in toxin, captopril and PHS groups these diferrences were significant (Figure 6 and Table 1). 

**Table1:** Total number of Nissl-stained neurons of SN on the left and right hemisphere.

SN	Control	Toxin	Captopril	*Peganum harmala*
Left (lesion side)	124.6 ± 4.2††	49.7 ± 9.0*	71.5 ± 2.7††	82.0 ± 5.3*††
Right (intact side)	130.8 ± 6.1	123.7 ± 10.7	135.3 ± 1.7	133.5 ± 12.0

Average (± SD) total number of Nissl-stained neurons in control, toxin, captopril and *Peganum harmala* groups two weeks after the lesion.

* *p *< 0.05 in comparison with its right hemisphere.

†† *p *< 0.01 in comparison with the toxin group on the left hemisphere.

## Discussion

We studied the protective effect of AEPHS on male rats with Parkinson's disease induced by 6-OHDA. Data showed AEPHS (10 mg/kg) improved movement criteria in diseased rats by lowering rigidity and apomorphine induced rotation. Oxidative stress markers such as lipid peroxidation and protein oxidation in the brain of *Peganum* group were significantly lower than toxin group. Brain ACE activity in the *Peganum* was significantly lower than toxin and control groups. In histology study, AEPHS group had more vital dopaminergic neurons compared to toxin group. These results were compared to captopril and showed that the crude extract of *Peganum* was more potent than pure captopril in reducing PD signs and oxidative stress markers. 

Many studies have shown that free radicals are destructive chemical substrates in PD (20-22). Imbalance between oxidant and antioxidant system can induce destructive effects of free radicals. Increasing in lipid peroxidation and decreasing antioxidants can induce PD (23). 6-OHDA is a catecholaminergic neurotoxin that is widely used as a laboratory chemical in PD model studies. Many data show that 6-OHDA has a close relationship with free radicals, because malondialdehyde increases in the presence of 6-OHDA (5, 24).

Captopril can reduce oxidative stress by 6-OHDA significantly and it is suggested that this ACE inhibitor can reduce dopaminergic neurodegeneration and progression of disease (12, 25). ACE inhibitors are efficient by scavenging ROS (26). Although some studies suggest that ACE inhibitors with "SH" group (like captopril) scavenge ROS, other studies show that this capacity is unrelated to "SH" group, and ACE inhibitors without "SH" group have the same antioxidant power (26, 27). This effect of ACE inhibitors may relate to prevention of angiotensin П synthesis (28). Angiotensin II induces oxidative stress in the brain by NADPH (12, 29). NADPH oxidase has distribution in brain (30, 31). Nontoxic doses of some neurotoxins can help to destruction of dopaminergic neurons related to NADPH, and production of ROS (23).

Brain angiotensin can promote dopaminergic degeneration and PD (28, 32), and blocking of this system could improve PD (11-13, 26, 33-36).

*Peganum harmala *L*.* is a full source of β carboline alkaloids. Some of its important alkaloids are harmine, harmaline, and harmalol (37). Harmaline inhibits ACE comparable to captopril (14). In a study of 135 herbal medicines for their ACE inhibitory effect, *Peganum harmala* showed a complete inhibition (15). β carboline alkaloids are benzodiazepine antagonists and inhibitors of amine oxidases, too (38). 

We showed that *Peganum harmala* extract had antioxidant and ACE inhibitory effect*. Peganum harmala* decreased lipid peroxidation and protein oxidation in the brain of rats with 6-OHDA induced PD, and increased vital neurons in SN, which improved PD symptoms. 

## Conclusion

 These findings demonstrate that *peganum harmala* L. has protective effect on 6-OHDA induced hemi-Parkinsonism rats, which might be through ACE inhibition.
